# TP53 to mediate immune escape in tumor microenvironment: an overview of the research progress

**DOI:** 10.1007/s11033-023-09097-7

**Published:** 2024-01-25

**Authors:** Kai-li Zhu, Fei Su, Jing-ru Yang, Ruo-wen Xiao, Rui-yue Wu, Meng-yue Cao, Xiao-ling Ling, Tao Zhang

**Affiliations:** 1 The First Clinical Medical College of Lanzhou University, Lanzhou, 730000 Gansu People’s Republic of China; 2 Department of Oncology, The First Hospital of Lanzhou University, Lanzhou, 730000 Gansu People’s Republic of China

**Keywords:** *TP53*, Tumors, Immune escape, Immunotherapy

## Abstract

Increasing evidence suggests that key cancer-causing driver genes continue to exert a sustained influence on the tumor microenvironment (TME), highlighting the importance of immunotherapeutic targeting of gene mutations in governing tumor progression. *TP53* is a prominent tumor suppressor that encodes the p53 protein, which controls the initiation and progression of different tumor types. Wild-type p53 maintains cell homeostasis and genomic instability through complex pathways, and mutant p53 (Mut p53) promotes tumor occurrence and development by regulating the TME. To date, it has been wildly considered that *TP53* is able to mediate tumor immune escape. Herein, we summarized the relationship between *TP53* gene and tumors, discussed the mechanism of Mut p53 mediated tumor immune escape, and summarized the progress of applying p53 protein in immunotherapy. This study will provide a basic basis for further exploration of therapeutic strategies targeting p53 protein.

## Introduction

Tumors are complex diseases involving multiple factors and stages. Immune escape plays a key role in tumorigenesis and tumor progression. The complex interaction between the immune system and tumor cells determines the tumor state through a process called “tumor immune editing,” which includes three phases: clearance, homeostasis and escape [1, 2]. In the immune clearance phase, the immune system recognizes, monitors, and removes most malignant cells; however, a small number of malignant cells survive and enter the homeostasis phase. At this stage, although continuous pressure from the adaptive immune system prevents tumor cell growth and expansion, tumor cells with genetic instability form immunogenically reduced tumor subclones under pressure, Finally, the tumor cells evade the antitumor immune mechanism and enter the immune escape stage [3, 4].The tumor microenvironment (TME) is a dynamic and complex environment where tumor cells arise, and its composition varies by tumor type.[5]. During the “tumor immune editing” process, each cellular and non-cellular component of the TME uniquely regulates tumor immune escape. Therefore, the goal of tumor immunotherapy is to counteract this immune escape by both maintaining the tumor-immune cycle in the TME and reactivating the anti-tumor immune response [6].

Specific genetic alterations in different types of cancer can influence tumor growth and metastasis in vivo by regulating the TME. The human melanoma model with progressive genome editing constructed by Regev et al. [7] revealed that the combination of tumor gene mutations can not only impact the cellular composition of the TME but also re-edit the cellular state of the individual cell types that constitute it, thus affecting tumorigenesis and progression. *TP53* mutations are prevalent in tumor progression. *TP53* encodes the p53 protein, which is a prominent core tumor suppressor [8]. As a transcription factor, p53 regulates the transcription of target genes by directly binding to p53 DNA-binding elements in their promoter regions [9]. wild-type p53 (WT p53) regulates cell apoptosis, cellular senescence, cell cycle arrest, DNA damage repair, metabolic adaptation, and other cellular stress responses to exert tumor-suppressive effects [10]. However, *TP53* missense mutations could disrupt the structural domain of the p53 protein, thereby impairing the expression or function of WT p53 and depriving it of its tumor-suppressive activity [11]. Tumor-derived mutant p53 (Mut p53) proteins contain missense, frameshift, truncation and deletion mutations; of which, approximately 74% are missense mutations mostly occurring within the p53 DNA-binding domain (DBD) [12]. Mut p53 has traditionally been classified as a “conformational” or “DNA contact” mutation. The former mainly interferes with folding of the core domain of p53 and is unable to bind to DNA or activate target genes. The latter directly mutates the amino acid residues that bind to DNA [13]. Both types of Mut p53 are unable to transactivate the target genes of WT p53 and therefore cannot mediate tumor-suppressive processes.

In addition to the loss of the tumor suppressor function of WT p53, Mut p53 promotes tumor progression through a gain-of-function (GOF) mechanism. To date, various Mut p53 GOF activities have been reported, including the promotion of tumor cell proliferation and metastasis, genomic instability, metabolic reprogramming, cell dryness, tumor microenvironment remodeling, immunosuppression and cancer treatment drug resistance [14]. Mut p53 usually exhibits different GOF activities through different molecular mechanisms. Further, Mut p53 forms complexes with the transcription factor NF-Y and cofactor p300 and transcribes and activates target genes of NF-Y, such as cyclin A, cyclin B1 and cyclin-dependent kinase 1 (CDK1), to promote cancer cell proliferation [15]. Specifically, most studies on the molecular mechanism of Mut p53 promoting metastasis have been found in rectal and pancreatic cancer cells. Mut p53^R248W^ stabilizes proteins through a heat shock protein 90 chaperone mechanism in pancreatic cancer and selectively binds to phosphorylated STAT3 in pancreatic cancer cells to form the Mut p53-pSTAT3 complex, which promotes tumor cell migration [16]. This evidence paves way for novel avenues for using Hsp90 inhibitors in patients with Mutp53 mutations. Notably, the metabolic changes in glucose, lipids and nucleotides are not only markers of tumor cells but are also key factors in tumor development [17]. Innate immune cells promote insulin resistance by secreting cytokines to regulate metabolism, reflecting the complex role between metabolism and immunity [18]. The Warburg effect is defined as a faster glucose uptake and lactic acid accumulation in tumor cells than in normal cells under aerobic conditions [19]. Mut p53 promotes the translocation of glucose transporter 1 to the cell membrane by activating RhoA, thereby promoting the Warburg effect in cancer cells [20]. Glycolysis and metabolism reshape the tumor microenvironment, and both computational and experimental analyses have shown that glycolysis increases PD-L1 expression in tumor [21]. Glycolytic lactic acid production can promote tumor cells and tumor-associated macrophages (TAMs) to secrete a series of factors supporting angiogenesis, whereas glucose deprivation and extracellular acidosis significantly inhibit the antitumor function of macrophages, CD4 + T cells, CD8 + T cells, and dendritic cells (DCs). However, they have little effect on immunosuppressive cells such as myeloid-derived suppressor cells (MDSCs) and regulatory T cells (Tregs), and cancer-associated fibroblasts (CAF) and tumor cells can promote glycolysis [22]. Therefore, tumors carrying Mut p53 protein may promote the transition from metabolism to glycolysis through the abovementioned mechanisms, leading to tumor progression. In addition, Mut p53 can increase the expression and activity of manganese superoxide dismutase, a key antioxidant detoxification enzyme, in melanoma cells through SIRT3-mediated deacetylation, which helps regulate the level of reactive oxygen species (ROS) and prevent its cytotoxicity [23]. Damages to the glycolysis pathway in tumor cells leads to the increase in ROS level, which downregulates c-FLIP, the key inhibitor of tumor necrosis factor-α-induced cell death and enhances CTLs-mediated bystander killing [24]. Therefore, the above results indicate a potential molecular mechanism of the Mut p53-induced glycolysis pathway in promoting tumor immune escape. Mevalonate pathway is important for lipid metabolism. Its metabolic intermediate mevalonate-5-phosphate (M5p) promotes the stability of Mut p53 by inhibiting the proteasomal degradation of Mut p53 mediated by the ubiquitin ligase CHIP, whereas Mut p53 binds to SREBP2 and promotes the mevalonate pathway to increase M5p levels, thus forming a positive feedback loop [25, 26]. In addition, Mut p53 binds to the ETS2 site in the target gene promoter to activate the expression of several nucleotide metabolic genes, such as dCK, TK1, and GMPS and promote nucleotide synthesis [27]. The activation of GMPS can mediate the production of inflammatory cytokines, INF-α, and tumor necrosis factor (TNF), thus activate STAT and NF-κB pathways in cancer cells, stimulate their growth, and increase chemotherapy resistance [28]. Therefore, to a large extent, Mut p53 achieves its GOF activity through metabolic reprogramming, thereby promoting tumor immune escape. Further studying the mechanism through which Mutp53 promotes tumor immune escape is crucial to identify potential antitumor therapeutic targets.

Recent studies have suggested that WT p53 protein plays an important role in the immune clearance phase of tumors. The Mut p53 can disrupt this function, thus affecting the tumor immune microenvironment [29]. Major histocompatibility complex-I (MHC-I)-mediated antigen presentation plays a key role in the antitumor adaptive immune response. WT p53 upregulates the expression of Tap1 and the aminotransferase Erap1, which are peptide transport proteins essential for MHC-I transport to the cell surface [30]. The expression of MHC-I on the cell surface decreased under *TP53* mutation conditions. In addition, Ghosh et al. [31] found that Mut p53 inhibits the function of the cGAS-STING-TBK1-IRF3 pathway, a cytoplasmic DNA-sensing mechanism in the natural immune response, by binding to TANK-binding kinase-1 (TBK1) and reducing the infiltration of lymphocytes, such as natural killer (NK) cells and CD8^+^ T cells, in the TME, thereby contributing to tumor immune escape. Therefore, Mut p53 may promote immune escape by inhibiting the antitumor immune response to form an immunosuppressive TME.

A review of related studies revealed that Mut p53 forms a tumor-promoting immunosuppressive microenvironment by damaging its function, reducing its number, and inhibiting the recruitment of immune cells to the TME [32–34]. In addition, Mut p53 has been reported to regulate the function of tumor stromal cells, other than immune cells, to promote tumor immune escape [35–37]. This review aimed to describe how Mut p53 regulates the immune landscape in the TME to create an ecological niche for tumor immune escape and summarize the current status of research on immunotherapy related to targeting the p53 pathway.

## Mut p53 mediates immune escape by regulating the immune cell component in TME

Mut p53-mediated tumor immune escape is dominated by tumor-induced immunosuppressive effects, resulting in an immunosuppressive TME [38]. The immune ecological niche of the TME is regulated by cytotoxic T lymphocytes (CTLs), NK cells, MDSCs, TAMs and Tregs. The abundance and composition of each immune cell type are different in different tumor types. Mut p53 can regulate the TME by affecting single-cell functions, such as reducing CTL and NK cell infiltration and inhibiting their antitumor activity while recruiting large numbers of immunosuppressive cells (MDSCs, TAMs and Tregs) into the TME, ultimately creating a pro-tumor immune ecological niche by controlling cytokine secretion.

### Mut p53 attenuates CTL cell-mediated antitumor immune response

**Fig. 1:**
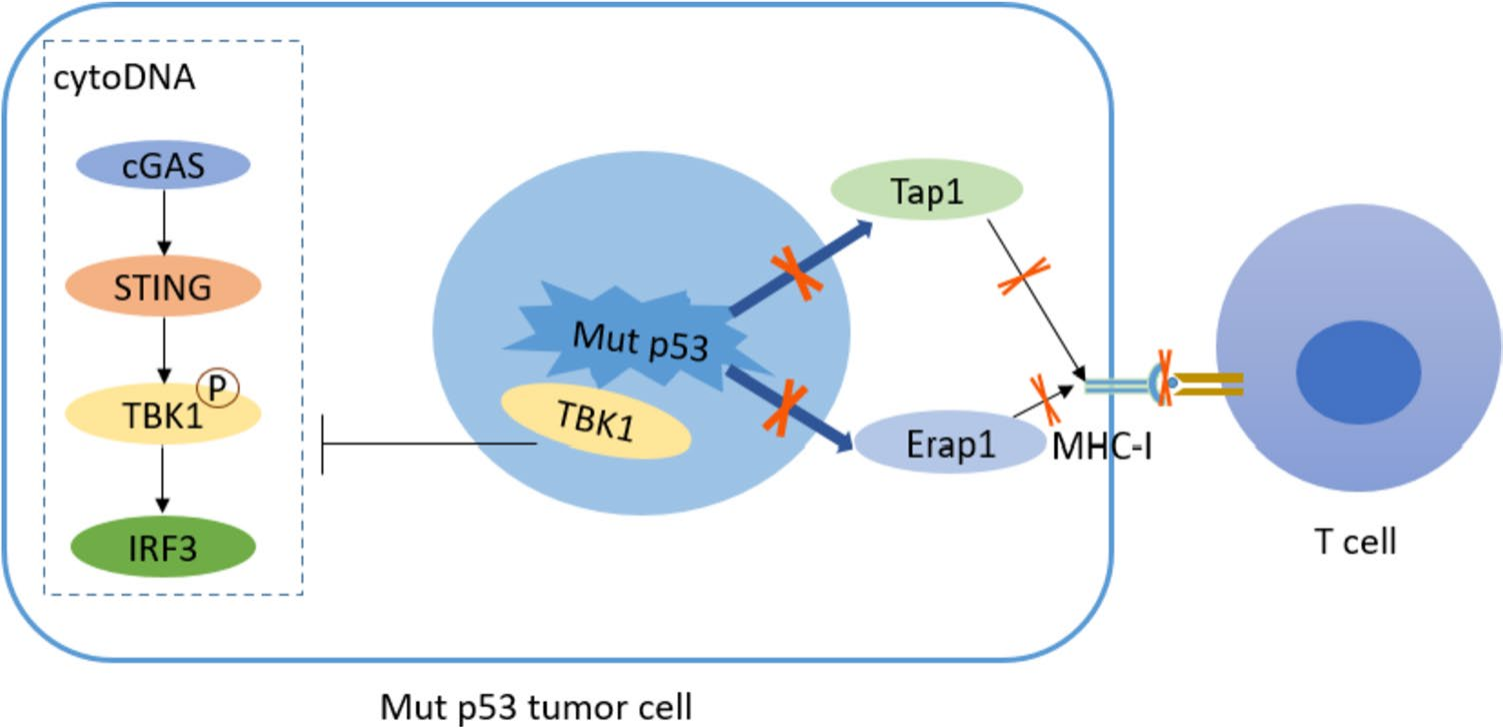
Schematic diagram of Mut p53 blocking anti-tumor immune response. Mut p53 down-regulates the expression of Tap1 and Erap1, which are necessary for MHC-I to be transported to the cell surface. The expression of MHC-I on the cell surface decreases and MHC-antigen complex decreases, which can not be recognized by T cells and finally blocks the anti-tumor adaptive immune response. On the other hand, Mut p53 inhibits TBK1 phosphorylation by binding with TBK1, thus inhibiting the function of cGAS-STING-TBK1-IRF3 pathway, which is a cytoplasmic DNA sensing mechanism in natural immune response, and reducing the infiltration of NK cells, CD8^+^T cells and other lymphocytes in TME, leading to tumor immune escape

CTLs are important effector cells involved in killing tumor cells. Recent studies have suggested that Mut p53 promotes tumor immune escape by disrupting CTL function or reducing the number of CTLs in the TME. Activated CTLs kill target tumor cells mainly through two pathways [39–41]: first, CTL release perforin (PFN) to transport secreted granzymes A and B (GzmA and GzmB) into tumor cells after forming pores in the tumor cell membrane, triggering an enzyme chain reaction that leads to apoptosis of tumor cells. Second, the death factor Fas ligand (FasL) on the surface of CTL binds to the Fas receptor (FasR) on the surface of tumor cells and activates cystatin 8 to initiate apoptosis. Studies have shown that GzmB can induce the accumulation of WT p53 in the mitochondria of target tumor cells and interact with the anti-apoptotic protein B-cell lymphoma-2 (BCL-2), thereby antagonizing the inhibitory effect of BCL-2 on the pro-apoptotic factors Bcl-2 Associated X-protein (BAX) and truncated Bid (tBid), contributing to a large release of BAX and tBid, thereby facilitating the release of cytochrome C from mitochondria and triggering the apoptosis of tumor cells [42]. Blocking the WT p53/BCL-2 interaction significantly reduced CTL/NK-mediated cytotoxicity of WT p53 target cells. Hence, further research is needed to determine whether Mut p53 is related to the resistance of tumor cells to PFN/GzmB-and CTL/NK-mediated cell death. Additionally, WT p53 positively regulates TNF-related apoptosis-inducing ligand-induced apoptotic pathways by directly upregulating FasR and death receptors 4 and 5 [43]. Mut p53 was also found to reprogram TNF-α signal transduction in tumor cells by binding tumor suppressor DAB2IP and downregulate its expression, that is, it promoted the activation of NF-κB and inhibited the activation of TNF-α on ASK1/JNK and finally promoted the survival of tumor cells and resisted the tumor killing effect mediated by CTL [44]. Mutp53 also activates Rac1, promotes AKT activation, and promotes the survival of tumor cells [45]. However, the mechanism through which AKT activation promotes the survival of tumor cells remains unclear and requires further investigation; therefore, Mut p53 may diminish the killing effect of CTL on tumor cells and promote tumor immune escape (Fig. 1).

It was also found that the number of bone marrow-infiltrating CTLs and T helper (Th) cells was reduced in patients with acute myeloid leukemia with Mut p53, whereas the number of Treg cells, which promote immune escape, was increased [46]. Mechanistically, Mut p53 reduced the number of CTLs and Th cells by downregulating the expression of the costimulatory molecule OX40 and increased the infiltration of Treg cells by upregulating the expression of the costimulatory molecule ICOS [47, 48]. In summary, Mut p53 may influence the immunosuppressive microenvironment by regulating immune cell function and numbers. Moreover, the reactivation of WT p53 function may be a novel approach to optimize CTL-mediated tumor killing.

### Mut p53 inhibits normal NK cell function

**Fig. 2:**
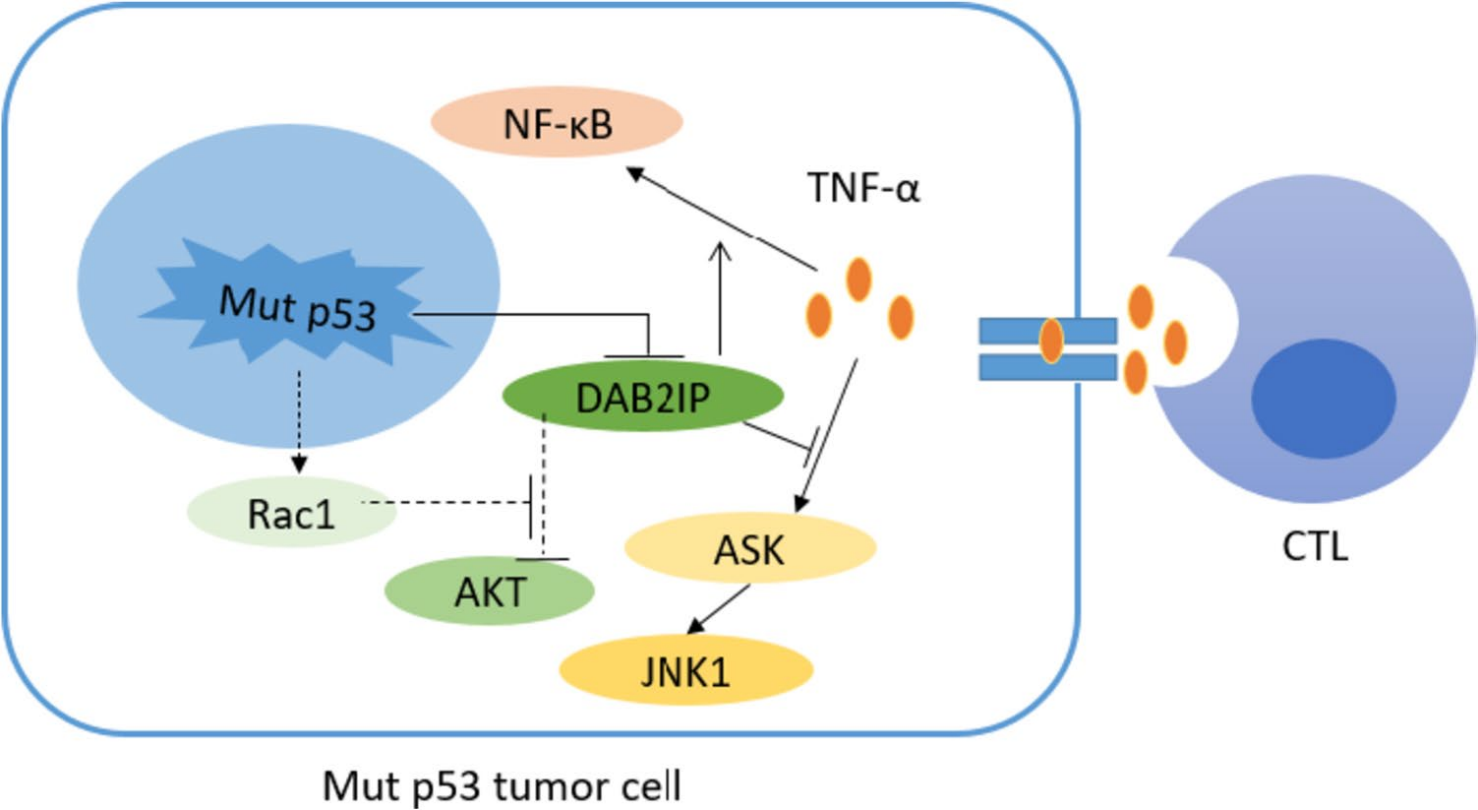
Schematic diagram of Mut p53 participating in regulating CTL cell-mediated anti-tumor immunity. Mut p53 reprogrammed the signal transduction of TNF-α in tumor cells by binding with tumor suppressor DAB2IP and down-regulating its expression, that is, it promoted the activation of NF-κB and inhibited the activation of TNF-α on ASK1/JNK at the same time, finally promoted the survival of tumor cells and resisted the tumor killing effect mediated by CTL. Mut p53 can also inhibit DAB2IP-AKT interaction by activating Rac1, promote AKT activation and lead to tumor cell survival

Activation of NK cells by effector molecules, such as interleukin (IL)-2, IL-12, IL-15 and IL-18. Downregulation of NK cell-inhibitory receptors (NK-IRs) or upregulation of NK cell-activating receptors (NK-ARs) can trigger the release of TNF-α and interferon (IFN)-γ from NK cells, which in turn exert antibody-dependent cell-mediated cytotoxic effects to kill tumor cells [49, 50]. NK cell-mediated tumor cell recognition and lysis is mainly dependent on the expression of NK-ARs NKG2D and DNAM-1. WT p53 upregulates the expression levels of ULBP1 and ULBP2, members of the ligand of the NKG2D (NKG2DL) family, on the surface of tumor cells and enhances the NK cell-mediated antitumor immune response [51]. Similarly, in oncogenic MYCN-amplified neuroblastoma, JQ1, a BET-bromo structural domain inhibitor of MYCN, downregulates c-MYC and p53 levels, which in turn downregulate NKG2DL expression and render neuroblastoma cells resistant to NK cell-mediated killing [52]. In addition, the glycolysis inhibitor dichloroacetate downregulates the expression of NKG2DL family members MICA, MICB, and ULBP-1 in tumor cells lacking WT p53 or expressing Mut p53, thereby mediating immune escape [53]. The above evidence suggests that Mut p53 inhibits the tumor-killing effect of NK cells, mainly by regulating the expression of stress ligands on the surface of tumor cells (Fig. 2). Therefore, increasing the number of NK cells, enhancing NK cell activity, and promoting NK cell infiltration have become major strategies for immunotherapy.

### Mut p53 regulates the cell proliferation and differentiation of TAMs

As the most widely distributed type of immune cells in the TME, TAMs are mainly derived from circulating monocytes, which would differentiate into TAMs after being recruited to the TME and are stimulated by various cytokines [54]. TAMs are a highly plastic class of mixed phenotype cells, and consistent with the specific differentiation of activated macrophages into M1 and M2 types, TAMs are also polarized into M1-like and M2-like TAMs. M1-like TAMs are primarily activated by IFN-γ, transforming growth factor-α (TGF-α) or granulocyte–macrophage [55]. They are activated by IFN-γ, TGF-α or granulocyte–macrophage colony stimulating factor (GM-CSF); express CD68, CD80 and CD86; and secrete IL-1β, IL-6 and chemokine CXCL9, which exert antitumor effects. M2-like TAMs are primarily activated by IL-10 and TGF-β, expressing CD163, CD204 and CD206, secrete IL-10, TNF, CCL17, among others, which exerting pro-tumor effects [56].

**Fig. 3:**
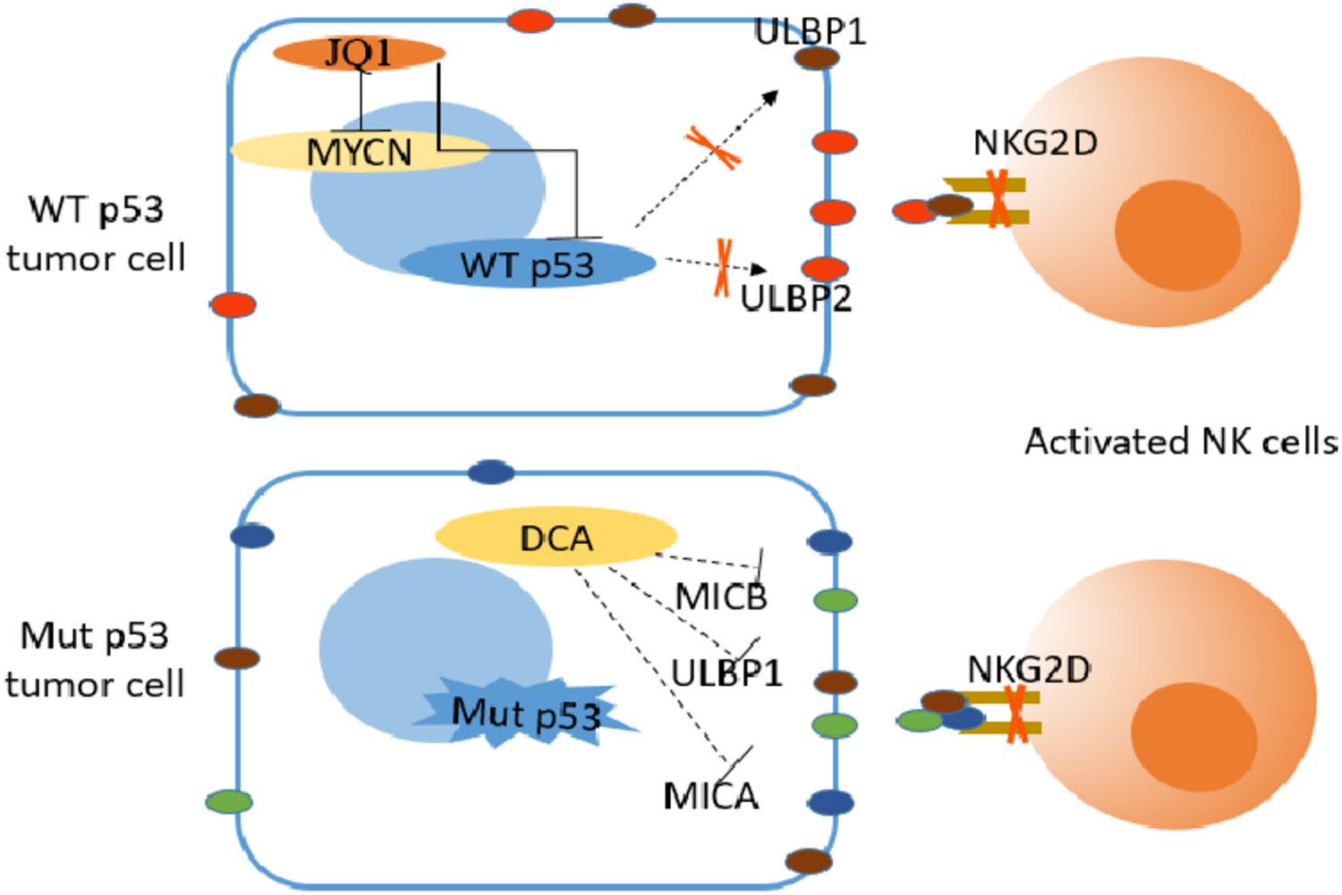
Schematic diagram of tumor cells lacking WT p53 or expressing Mut p53 against NK cell killing. WT p53 can directly up-regulate the expression levels of ULBP1 and ULBP2, members of the ligand family of NKG2D on the surface of tumor cells and enhance the anti-tumor immune response mediated by NK cells. JQ1, a BET-bromine domain inhibitor of oncogene MYCN, can down-regulate the levels of c-MYC and p53, and then down-regulate the expression of NKG2DL, making tumor cells resistant to NK cell-mediated killing. Dichloroacetate (DCA), a glycolytic inhibitor, can also down-regulate the expression of MICA, MICB and ULBP-1 of NKG2DL family in tumor cells lacking WT p53 or expressing Mut p53, thus mediating immune escape

In a mouse model of colorectal cancer, alterations in p53 function affect the proliferation of TAMs and regulate macrophage polarization. First, p53 affects the recruitment of TAMs in primary colorectal cancer cells. The intrinsic mechanism is that p53 inhibits colony-stimulating factor 1 receptor (CSF1R) expression by inducing miR-34a, which further inhibits the first step of the STAT3-mediated tumor cell metastatic cascade epithelial–mesenchymal transformation and promotes tumor cell migration [57]. In contrast, the high expression of CSF1R caused by TP53 mutation is considered to affect both the differentiation and proliferation of TAMs. The overexpression of NAD-dependent deacetylase sirtuin 1 (SIRT1) in colorectal cancer promoted the expression of CXCL12 and CXCR4 on the surface of tumor cells and macrophages, respectively, facilitated the recruitment of M2-like TAMs and inhibited the cell proliferation and activity of CD8 + T cells, thus promoting colorectal cancer progression [58]. Simultaneously, Mut p53 colorectal cancer cells selectively release miR-1246-rich exosomes, and uptake of these exosomes by macrophages triggers miR-1246-dependent self-reprogramming, resulting in a pro-tumor state phenotype [59]. Using a set of 16 different genetically engineered mouse models of breast cancer, Wellensten et al. [60] revealed that p53-deficient tumor cells can induce WNT-secreted ligands, increase circulating neutrophils and stimulate TAMs to produce IL-1β, thereby triggering CXCR4 systemic inflammation and leading to breast cancer cell metastasis. In summary, loss of function or mutation of *TP53* reprogrammed TAMs to promote tumor immune escape through different regulatory mechanisms (Fig. 3).

### *TP53* regulates the cell proliferation and differentiation of immunosuppressed cells

**Fig. 4:**
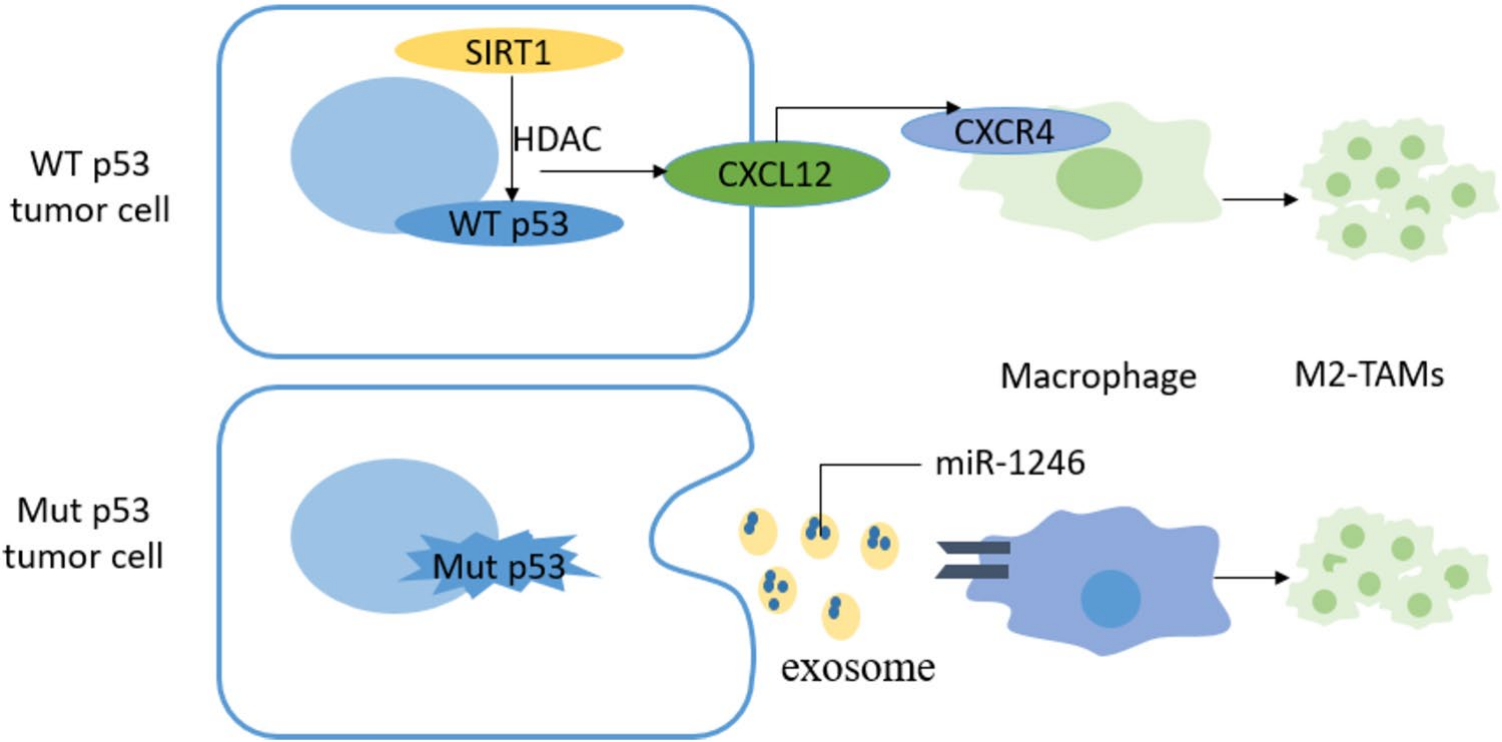
Schematic diagram of Mut p53 reprogramming TAMs. Over-expressed histone deacetylase SIRT1 in tumor cells promotes the expression of CXCL12 on the surface of tumor cells, promotes the high expression of CXCR4 on the surface of macrophages, and promotes the recruitment of M2-like TAMs. Mut p53 tumor cells selectively release exosomes rich in miR-1246. Macrophages ingest these exosomes and trigger miR-1246-dependent self-reprogramming, forming M2-like TAMs

MDSCs, which are a diverse group of myeloid-like cells in the TME, play a crucial role in anti-tumor activities by suppressing CTL activation [61]. The Fas–FasL cell death pathway was initially identified as a key regulator of CTL activity, and Fas-mediated apoptosis has been found to regulate MDSC homeostasis. In human colon cancer cells, activation of p53 upregulates Fas on the surface of MDSCs, which increases the sensitivity of MDSCs to FasL-induced cell apoptosis, that is, by activating the intrinsic p53–Fas–FasL pathway of MDSCs, promotes cell apoptosis, and reactivates CTL-mediated antitumor immunity [62]. DLEC1, a tumor suppressor linked to p53, interacts with the oncogenic signaling molecule, STAT3, in response to IL-6 stimulation, blocks the JAK2/STAT3 signaling pathway, and inhibits STAT3 phosphorylation, thereby controlling tumor progression [63]. Although Katz et al. [64] found that the GM–CSF/JAK2/STAT3 axis drives the cell proliferation of liver-associated MDSCs, and inhibition of STAT3 activates cell apoptotic signaling pathways in MDSCs, including the upregulation of the pro-apoptotic factor Bax and downregulation of the expression of the anti-apoptotic factor BCL-2. Thus, Mut p53 induces the cell proliferation by promoting STAT3 phosphorylation, leading to tumor immune escape. Ferroptosis is an iron,and ROS-dependent regulation of cell death induced by the degradation of heme oxygenase 1 (Hmox1), a downstream molecule of p53, and the release of free iron to produce ROS in the mitochondrial membrane [65]. Reportedly, ASAH2, a molecule highly expressed on the surface of tumor cells in a mouse colon cancer model, was also upregulated on the surface of MDSCs in TME and affected ferroptosis to promote the survival and accumulation of MDSCs by inhibiting the p53-Hmox1 pathway, consistent with the finding that silencing p53 reduced ROS levels in MDSCs and inhibited ferroptosis in MDSCs [66]. Thus, Mut p53 leads to the accumulation of immunosuppressive cellular MDSCs in the TME, mainly by blocking the apoptotic signaling pathway of MDSCs (Fig. 4).

Treg cells, an immunosuppressive subset of CD4 + T cells, primarily ensure immune balance and self-tolerance. They are abundant in the TME and inhibit CTL-driven anti-tumor responses.Currently, there are fewer studies on the mechanisms through which p53 regulates Tregs to influence antitumor immunity; however, in recent years, increasing evidence has suggested that Mut p53 promotes tumor immune escape by affecting Treg cell differentiation. p53 interacts with non-coding RNAs (lncRNAs and miRNAs) to regulate tumor immune escape. LncRNA MEG3 suppresses tumor immune escape by upregulating the expression of miR-149-3p through MDM2-mediated p53 and decreasing the expression of FOXP3, which ultimately reduces the differentiation and maturation of Treg cells [67]. In addition, miR-34, which is positively regulated by p53, can take part in the feedback loop of TGF-β. Elevated TGF-β activity inhibits miRNA-34a expression, leading to enhanced production of chemokine CCL22, which recruits Treg cells to promote immune escape [68]. p53 deficiency in prostate, ovarian, and pancreatic cancers increases the number of Tregs in TME. In conclusion, Mut p53 can promote tumor immune escape by regulating Treg cell differentiation and recruiting Treg cells, and immunotherapy targeting Mut p53 and Treg cells plays an essential role in suppressing tumor immune escape.

In summary, Mut p53 mediates tumor immune escape by regulating immune cells through different mechanisms in different tumor types, providing a basis for the development of different immunotherapeutic strategies. However, Mut p53 ultimately promotes tumor immune escape by regulating the secretion of different cytokines by tumor and immune cells, making it an immunosuppressive signaling molecule. The literature sources of the molecules involved in the above mechanisms are shown in Table 1.

**Table 1:** The action mechanism of some molecules described in this paper and its literature sources

Immune cells regulated by Mut p53	Mutp53-regulated protein	Regulation by Mut p53	Fuction	References
CTL cell	BCL-2	Interact with BCL-2	Pro-cancer: can reduced GzmB-mediated CTL killing	Ben Safta et al. [42]
	DAB2IP	Down-regulating DAB2IP	Pro-cancer: can promote the activation of NF-κB and inhibited the activation of TNF-α on ASK1/JNK, and finally promoted the survival of tumor cells	Di Minin et al. [44], Sorrentino et al. (2022)[112]
	OX40	Down-regulating OX40	Pro-cancer:can reduce the number of CTL and Th cells	Buchan et al. [47]
	ICOS	Up-regulating ICOS	Pro-cancer: can increase the infiltration of Treg cells	Amatore et al. [48]
NK cell	ULBP1/2	Down-regulating ULBP1/2	Pro-cancer:can attenuat NK cell-mediated anti-tumor immune responses	Duan et al. [51]
MDSCs cell	STAT3	Promote STAT3 phosphorylation	Pro-cancer:can induce the cell proliferation of MDSCs	Li et al. [51], Guha et al. [64]
	ROS	Down-regulating ROS	Inhibit ferroptosis in MDSCs	Chang et al. [65], Zhu et al. [66]
TAMs cell	miR-1246	Up-regulating miR-1246	Pro-cancer:can promote polarization of M2 macrophages	Cooks et al. [59]
	CSF1R	Up-regulating CSF1R	Pro-cancer: can promote the first step of STAT3-mediated tumor cell metastatic cascade epithelial-mesenchymal transformation	Shi et al. [57]
	IL-1β	Up-regulating IL-1β	Trigger CXCR4 systemic inflammation and lead to breast can Pro-cancer: can cer cell metastasis	Wellenstein et al. [60]
Treg cell	miR-149-3p	Down-regulating miR-149-3p	Pro-cancer: can promote Treg cell differentiation and maturation	Xu et al. [67]
	miR-34	Down-regulating miR-34	Pro-cancer: can recruit Treg cells to promote immune escape	Yang et al. [68]

## Mut p53 promotes immune escape by regulating non-immune cell components

In the TME, CAFs are the main components of the tumor stroma and exert immunosuppressive effects by secreting growth factors, extracellular matrix proteins and inflammatory ligands, thereby promoting non-restrictive tumor cell growth, angiogenesis and therapeutic resistance [69]. Mut p53 can affect tumor progression by altering the activity and function of CAFs. Mut p53 binds to STAT3 to promote STAT3 phosphorylation and upregulates the expression of α-smooth muscle actin, fibroblast-derived factor 10 and CXCL12 to activate CAFs [70]. CAFs of Mut p53 significantly affect the composition and function of immune cells in the TME by secreting cytokines, including CXCL12, stromal cell-derived factor 1 (SDF-1) and IL-6, mainly manifesting tumor-suppressive effects [71]. In addition, in colorectal cancer, ROS produced by mutant p53 tumor cells facilitate CAF-secreted vascular endothelial growth factor to regulate angiogenesis and ultimately promote tumor growth [72]. Therefore, Mut p53 promotes tumor immune escape by activating CAFs and regulating their secretion of growth factors. In recent years, CAFs have emerged as novel targets for cancer therapy, and mutated p53, a key regulator of CAF activation, has shown potential as a key pathway for novel therapeutic interventions. mesenchymal stem cells (MSCs) are also important cellular components of the TME that contribute to tumor growth and metastasis [73]. Mut p53 promotes tumor growth and proliferation by recruiting MSCs to the TME through a CXCL12-dependent mechanism [74]. Meanwhile, MSCs with Mut p53 are considered the cells of origin of bone tumors, affecting osteogenic differentiation and influencing the properties of osteosarcoma TME components such as inducible nitric oxide synthase, CCL5, IL-6 and TGF-β expression at higher levels, ultimately promoting osteosarcoma development [75].

In summary, Mut p53 induces the production of an immunosuppressive TME by promoting the secretion of various cytokines by non-immune cells in the TME, interacting with tumor cells, and ultimately promoting immune escape.

## Strategies for targeting Mut p53

The current mechanism of therapeutic action targeting Mut p53 involves the activation or restoration of WT p53 function in tumor cells. As mentioned above, Mut p53 forms an immunosuppressive microenvironment through different pathways. Therefore, WT p53 reactivation in the TME represents a promising therapeutic strategy to reverse immunosuppression and reshape the immunological landscape to support antitumor immunity.

### p53-based vaccines and specific antibodies

P53-derived peptides have been investigated as targets for various immunotherapeutic strategies, including vaccines, bipotent antibodies and TCR-like antibodies. Researchers have tested a modified cowpox virus (MVA) vaccine encoding WT p53 in combination with gemcitabine in patients with platinum-resistant ovarian cancer, and five of eleven subjects were able to induce CD8 + and CD4 + T cell responses [76]. In addition, studies on the p53 MVA vaccine in combination with immune checkpoint inhibitors, including PD-1/PD-L1 and CTLA-4 antibodies, are also underway. Although the vaccine was safe, induced an anti-Mut p53 immune response and achieved disease stabilization in some patients, there were no clinically significant benefits. Bispecific antibodies are innovative cancer immunotherapies with dual specificity for tumor antigens and TCR-CD3 complexes [77]. A Mut p53-based bispecific antibody recognizes neoantigens from the TP53 R175H mutation site and TCR-CD3 complex, overcoming the lack of neoantigen presentation and selectively redirecting T cells to recognize Mut p53 tumor cells [78]. Because of its intracellular localization p53, it is not recognized by classical therapeutic antibodies. TCR-like antibodies mimic the ability of T cells to recognize MHC I-presenting peptides and are specific to pMHCs that present WT and Mut p53 antigens [79]. TCR-like antibodies have shown promising in vitro and in vivo antitumor effects in animal models [80]. In conclusion, most vaccines and antibodies against Mut p53 are currently only validated in animal models but provide avenues for future tumor therapy. Simultaneously, current antibody therapies have overcome the previous limitation of not recognizing intracellular antigens, which provides new evidence for further development of antibodies targeting solid tumors.

### Molecular delivery targeting Mut p53

Although p53-based vaccines and specific antibodies have shown antitumor activity, they do not show clinical benefits; therefore, therapeutic strategies for Mut p53 proteins in tumor cells remain limited. Identifying effective and safe therapeutic strategies for Mut p53 is of great significance. Marco et al. [81] found that the delivery of oligonucleotides through modified nanomaterials can overcome the cancer resistance of refractory tumors carrying Mut p53 apoptosis because these nanostructures can inhibit the mTOR signaling pathway and anti-apoptotic protein Bcl-2. Resistance to temozolomide in the treatment of glioblastoma (GBM) is related to the upregulation of O6-methylguanine-DNA methyltransferase (MGMT), whereas the expression of MGMT in tumor cells is negatively regulated by WT p53 [82–84]. Researchers have developed a systemic nanodelivery platform (scL) for tumor-specific targeting that can deliver WT p53 through the blood–brain barrier, effectively target GBM and cancer stem cells (CSC), downregulate MGMT and induce GBM cell apoptosis. Simultaneously, the combination of scL-p53 and TMZ increased the antitumor efficacy of TMZ [85]. Therefore, scLs may provide a novel antitumor therapeutic approach that targets the delivery of molecules. In addition, researchers have developed a redox-responsive nanoparticle (NP) platform that can effectively deliver p53-encoded mRNA to induce cell cycle arrest and apoptosis, significantly delay the proliferation of p53-null hepatocellular carcinoma and non-small cell lung cancer cells, and considerably improve the sensitivity of tumor cells to mTOR inhibitors [86]. The mechanism of NP-mediated p53 gene delivery to decrease tumor progression is to inhibit tumor angiogenesis [87]. KRAS-TP53 co-mutation is closely related to poor prognosis of gastrointestinal tumors. Researchers have proposed a novel double-targeted HA-TPP/A nanocomplex that leads to the ubiquitin-dependent proteasomal degradation of Mut p53 by targeting mitochondrial damage, destroying its GOF activity, increasing the sensitivity of AMG510-induced tumor cell killing, and thus reducing the proliferation and migration of gastrointestinal cancer cells with KRAS-TP53 co-mutation [88]. Therefore, the delivery of molecular-targeted Mutp53 through nanocomposites has become a novel antitumor therapeutic approach, and the study of combined drug transformation based on its different mechanisms will also be focused on further improving the antitumor efficacy.

### WT p53 reactivation synergistic immunotherapy

Currently, combination drug therapy is an effective antitumor treatment strategy. In response to the variability in immune cell abundance in different tumor cells, it is crucial to explore immunotherapy for WT p53 reactivation in combination with other effective immunotherapeutic approaches to enhance immune cell recruitment and maintain the normal function of antitumor immune cells. Several p53 activators have been reported, but most studies are in the preclinical stage owing to the lack of specificity. The ubiquitin ligase mouse double minute 2 homolog (MDM2) is a major negative regulator of p53 and closed-loop negative feedback regulation of MDM2-p53 in normal cells maintains a dynamic balance between MDM2 and p53 expression levels [89]. Data from cancer cell lines, a cholera mouse model, and patients with melanoma suggest that MDM2 inhibitor-mediated pharmacological p53 reactivation triggers the ERV-dsRNA-IFN pathway in tumor cells, thereby altering the TME to a therapeutically responsive phenotype and triggering tumor immune surveillance [90]. Immune checkpoint PD-L1 on the surface of tumor cells binds to PD-1 on the surface of CTL to inhibit CTL activation and induce tumor immune escape [91]. The MDM2 inhibitor HDM201 triggered antitumor adaptive immunity in WT p53 tumors alone and further enhanced adaptive immunity by blocking the PD-1/PD-L1 pathway [92]. It suggests that WT p53 is required for the antitumor immune response, which explains the potential mechanism of MDM2 inhibitor combined with immune checkpoint inhibitor therapy. In addition, the team of professors Duda DG and Shi J jointly developed an mRNA tumor-targeting nanoparticle strategy to restore the function of WT p53, which inhibited the proliferation of Mut p53 hepatocellular carcinoma cells while inhibiting tumor growth in combination with anti-PD-1 therapies and significantly enhanced antitumor immunity in hepatocellular carcinoma [93]. This finding reaffirms the importance of WT p53 activators in combination with PD-1/PD-L1 antibodies against tumors. Among the patients with diffuse large B-cell lymphoma receiving CD19 chimeric antigen receptor (CAR) therapy, those with Mut p53 have poorer overall survival rates than those with WT p53; therefore, TP53 is a valuable prognostic biomarker [94]. This suggests that the impact of TP53 abnormalities on CAR-T cell therapy and the need for further research on the related signaling pathways should be considered when assessing the likelihood of success of CAR-T therapies in future and designing the clinical trials for high-risk patients.

In conclusion, Mut p53 can affect the immune ecological site of the TME, WT p53 expression is beneficial for immunotherapy, and the current therapeutic strategy for WT p53 reactivation in synergistic immunotherapy has shown promising antitumor efficacy in animal experiments. Therefore, combining small-molecule drugs that restore WT p53 activity with immunotherapy for tumor patients may improve the success rate of antitumor therapy.

## Discussion

At present, the TME, which is composed of the ECM, stromal cells, immune cells and blood vessels, plays a key role in tumor development and chemotherapy resistance [95]. It is usually associated with the evasion of the immune system. The loss of function of WT p53 is key to the formation of an immunosuppressive microenvironment, which not only suppresses the antitumor immune response but is also beneficial to the proliferation of tumor cells [33]. Mut p53 can produce not only a proinflammatory but also an antiinflammatory environment. Mut p53 subtypes can have a profound impact on gene expression patterns, many of which lead to tumor cell proliferation and chemotherapy resistance in various ways [96]. Mut p53 regulates immune escape by increasing the expression of programmed death ligand 1 (PD-L1) in tumor cells [97]. Mut p53 decreases the expression of miR-34/miR-200 during mesenchymal transformation, resulting in an increase in PD-L1 expression in NSCLC cells [98]. Higher PD-L1 levels show significant benefits for PD-1/PD-L1 blocking therapy, highlighting Mut p53 as a potential target for immunotherapy. In cervical cancer, PD-L1 levels can be increased by miR-18a by targeting SOX6 to activate the Wnt/β-catenin pathway and inactivate p53 signaling [99]. Therefore, immune checkpoint inhibitors have potential efficacy in Mut p53 tumors. In addition, inflammation may promote the occurrence and development of cancer and induce the formation of immunosuppressive microenvironment, which is affected by the coordination of several proinflammatory cytokines and carcinogenic pathways, which include NF-kB, MAPK, mTOR and STAT3 signaling pathways. These carcinogenic pathways promote the stability of Mut p53, thus blocking the interaction between the carcinogenic pathways and proinflammatory cytokines to reduce carcinogenic inflammation and Mut p53 expression [100]. Therefore, inhibitors targeting these carcinogenic pathways, such as AG490 and WP1066 [101, 102], mTOR inhibitors [103] targeting the STAT3 pathway, LY3007113 [104] targeting p38MAPK and 17-AAG [105] targeting HSP90, may have promising antitumor efficacy alone or in combination. Many of these inhibitors are already in preclinical or clinical trials and have shown promising results. In addition, patients with Mut p53 appear to be more sensitive to immunosuppressant therapy. Mut p53 can affect immune cell infiltration, cytokine secretion, and inflammatory pathways in the TME and thus significantly affect the antitumor immune response. Mutated p53 cells increase the load of novel antigens and improve the inhibitory response to immune checkpoints. The recovery of WT p53 can induce an antitumor immune response in immune cold tumors, but antitumor immune activation and tumor regression of Mut p53 are heterogeneous in cancer types [106]. At present, other immunotherapies such as adoptive cell therapy, monoclonal antibodies, oncolytic viruses, and immune system regulators have shown efficacy in various types of cancer; however, their efficacy needs to be explored in combination with WT p53 reactivation therapy. In summary, exploring the mechanism through which Mut p53 promotes tumor immune escape is highly significant to further improve the efficacy of antitumor immunity.

Cell-to-cell interactions are crucial for cellular communication; therefore, choosing appropriate tools to evaluate the role of p53 is a prerequisite for exploring the mechanism of tumor immune escape promoted by Mutp53. Bulk RNA-sequencing (RNA-seq), single-cell RNA sequencing (scRNA-seq), and other RNA-seq techniques have emerged to reveal transcriptome heterogeneity. Bulk RNA-sequencing involves transcriptome sequencing of a large number of mixed cells; however, this method could only obtain the average level of gene expression [107]. ScRNA-seq enables the study of gene expression at the single-cell level and has shown advantages in discovering novel cell types and revealing cell heterogeneity; however, single-cell sequencing has lost tissue spatial location information [108]. The latest developments in the spatial transcriptome (ST) can indicate both the level of gene expression and the spatial location of cells [109]. Researchers have tested 16 cell–cell interaction methods by combining scRNA-seq and ST data and have shown that the interactions predicted using different tools are highly random [110]. This tool, named CellTrek, can map single cells to the spatial coordinates of tissue sections according to scRNA-seq and ST data, and study single-cell data with spatial information more flexibly and directly [111].

In summary, there is a very high probability of *TP53* mutations in tumors. We described the specific mechanism through which Mut p53 mediates tumor immune escape by forming an immunosuppressive microenvironment, highlighting the key tumor-suppressive role of p53. p53 is considered an important target for antitumor immunotherapy, and various small-molecule drugs have been developed. Targeting Mut p53 therapy in combination with other immunotherapeutic approaches is also being explored. However, many questions remain unanswered, such as the types of TP53 mutations and factors affecting their mutation profiles, the specific mechanisms regulating *TP53* mutations, and the mechanisms through which the p53 pathway interacts with other pathways to affect tumor progression. Currently, therapeutic agents targeting Mut p53 are under experimental and clinical investigations. In addition to drug combination therapy, WT p53 drug resistance, complex changes within tumor cells after drug administration to cope with off-target effects and toxicity, and interspecies variability between animals and humans leading to the need for in vitro models to verify drug efficacy are all issues that need attention in future research.

## Data Availability

No Data associated in the manuscript.

## References

[1] O’Donnell JS, Teng MWL, Smyth MJ (2019) Cancer immunoediting and resistance to T cell-based immunotherapy. Nat Rev Clin Oncol 16(3):151–167 30523282 10.1038/s41571-018-0142-8

[2] Lei X, Lei Y, Li JK et al (2020) Immune cells within the tumor microenvironment: biological functions and roles in cancer immunotherapy. Cancer Lett 470:126–133 31730903 10.1016/j.canlet.2019.11.009

[3] Riaz N, Havel JJ, Makarov V et al (2017) Tumor and microenvironment evolution during Immunotherapy with Nivolumab. Cell 171(4):934–49.e16 29033130 10.1016/j.cell.2017.09.028 PMC5685550

[4] Wu Q, Wu W, Franca TCC et al (2018) Immune evasion, a potential mechanism of Trichothecenes: new insights into negative immune regulations. Int J Mol Sci 19(11):3307 30355984 10.3390/ijms19113307 PMC6275004

[5] Arneth B (2019) Tumor microenvironment. Medicina 56(1):15 31906017 10.3390/medicina56010015 PMC7023392

[6] Rui R, Zhou L, He S (2023) Cancer immunotherapies: advances and bottlenecks. Front Immunol 14:1212476 37691932 10.3389/fimmu.2023.1212476 PMC10484345

[7] Hodis E, Torlai Triglia E, Kwon JYH et al (2022) Stepwise-edited, human melanoma models reveal mutations’ effect on tumor and microenvironment. Science 376(6592):eabi8175 35482859 10.1126/science.abi8175 PMC9427199

[8] Kaur RP, Vasudeva K, Kumar R et al (2018) Role of p53 gene in breast cancer: focus on mutation spectrum and therapeutic strategies. Curr Pharm Des 24(30):3566–3575 30255744 10.2174/1381612824666180926095709

[9] Mantovani F, Collavin L, Del Sal G (2019) Mutant p53 as a guardian of the cancer cell. Cell Death Differ 26(2):199–212 30538286 10.1038/s41418-018-0246-9 PMC6329812

[10] Brown K, Jenkins LMM, Crooks DR et al (2022) Targeting mutant p53–R248W reactivates WT p53 function and alters the onco-metabolic profile. Front Oncol 12:1094210 36713582 10.3389/fonc.2022.1094210 PMC9874945

[11] Chen X, Zhang T, Su W et al (2022) Mutant p53 in cancer: from molecular mechanism to therapeutic modulation. Cell Death Dis 13(11):974 36400749 10.1038/s41419-022-05408-1 PMC9674619

[12] Zhou X, Hao Q, Lu H (2019) Mutant p53 in cancer therapy-the barrier or the path. J Mol Cell Biol 11(4):293–305 30508182 10.1093/jmcb/mjy072 PMC6487791

[13] Walerych D, Lisek K, Del Sal G (2015) Mutant p53: one, no one, and one hundred thousand. Front Oncol 5:289 26734571 10.3389/fonc.2015.00289 PMC4685664

[14] Zhang C, Liu J, Xu D et al (2020) Gain-of-function mutant p53 in cancer progression and therapy. J Mol Cell Biol 12(9):674–687 32722796 10.1093/jmcb/mjaa040 PMC7749743

[15] Di Agostino S, Sorrentino G, Ingallina E et al (2016) YAP enhances the pro-proliferative transcriptional activity of mutant p53 proteins. EMBO Rep 17(2):188–201 26691213 10.15252/embr.201540488 PMC5290815

[16] Klemke L, Fehlau CF, Winkler N et al (2021) The gain-of-function p53 R248W mutant promotes migration by STAT3 deregulation in human pancreatic cancer cells. Front Oncol 11:642603 34178628 10.3389/fonc.2021.642603 PMC8226097

[17] Kreuzaler P, Panina Y, Segal J et al (2020) Adapt and conquer: metabolic flexibility in cancer growth, invasion and evasion. Mol Metab 33:83–101 31668988 10.1016/j.molmet.2019.08.021 PMC7056924

[18] Lackey DE, Olefsky JM (2016) Regulation of metabolism by the innate immune system. Nat Rev Endocrinol 12(1):15–28 26553134 10.1038/nrendo.2015.189

[19] Liberti MV, Locasale JW (2016) The Warburg effect: how does it benefit cancer cells? Trends Biochem Sci 41(3):211–218 26778478 10.1016/j.tibs.2015.12.001 PMC4783224

[20] Zhang C, Liu J, Liang Y et al (2013) Tumour-associated mutant p53 drives the Warburg effect. Nat Commun 4:2935 24343302 10.1038/ncomms3935 PMC3969270

[21] Jiang Z, Liu Z, Li M et al (2019) Increased glycolysis correlates with elevated immune activity in tumor immune microenvironment. EBioMedicine 42:431–442 30935888 10.1016/j.ebiom.2019.03.068 PMC6491961

[22] Zhong X, He X, Wang Y et al (2022) Warburg effect in colorectal cancer: the emerging roles in tumor microenvironment and therapeutic implications. J Hematol Oncol 15(1):160 36319992 10.1186/s13045-022-01358-5 PMC9628128

[23] Torrens-Mas M, Cordani M, Mullappilly N et al (2020) Mutant p53 induces SIRT3/MnSOD axis to moderate ROS production in melanoma cells. Arch Biochem Biophys 679:108219 31812668 10.1016/j.abb.2019.108219

[24] Wu L, Jin Y, Zhao X et al (2023) Tumor aerobic glycolysis confers immune evasion through modulating sensitivity to T cell-mediated bystander killing via TNF-α. Cell Metab 35(9):1580–96.e9 37506695 10.1016/j.cmet.2023.07.001

[25] Juarez D, Fruman DA (2021) Targeting the mevalonate pathway in cancer. Trends Cancer 7(6):525–540 33358111 10.1016/j.trecan.2020.11.008 PMC8137523

[26] Parrales A, Thoenen E, Iwakuma T (2018) The interplay between mutant p53 and the mevalonate pathway. Cell Death Differ 25(3):460–470 29238070 10.1038/s41418-017-0026-y PMC5864191

[27] Schmidt V, Nagar R, Martinez LA (2017) Control of nucleotide metabolism enables mutant p53’s oncogenic gain-of-function activity. Int J Mol Sci 18(12):2759 29257071 10.3390/ijms18122759 PMC5751358

[28] Chen Q, Boire A, Jin X et al (2016) Carcinoma-astrocyte gap junctions promote brain metastasis by cGAMP transfer. Nature 533(7604):493–498 27225120 10.1038/nature18268 PMC5021195

[29] Gomes AS, Ramos H, Inga A et al (2021) Structural and drug targeting insights on mutant p53. Cancers 13(13):3344 34283062 10.3390/cancers13133344 PMC8268744

[30] Wang B, Niu D, Lai L et al (2013) p53 increases MHC class I expression by upregulating the endoplasmic reticulum aminopeptidase ERAP1. Nat Commun 4:2359 23965983 10.1038/ncomms3359 PMC3759077

[31] Ghosh M, Saha S, Bettke J et al (2021) Mutant p53 suppresses innate immune signaling to promote tumorigenesis. Cancer Cell 39(4):494-508.e5 33545063 10.1016/j.ccell.2021.01.003 PMC8044023

[32] Asl ER, Rostamzadeh D, Duijf PHG et al (2023) Mutant P53 in the formation and progression of the tumor microenvironment: friend or foe. Life Sci 315:121361 36608871 10.1016/j.lfs.2022.121361

[33] Blagih J, Buck MD, Vousden KH (2020) p53, cancer and the immune response. J Cell Sci. 10.1242/jcs.237453 32144194 10.1242/jcs.237453

[34] Agupitan AD, Neeson P, Williams S et al (2020) P53: a guardian of immunity becomes its saboteur through mutation. Int J Mol Sci 21(10):3452 32414156 10.3390/ijms21103452 PMC7278985

[35] Stein Y, Aloni-Grinstein R, Rotter V (2019) Mutant p53-a potential player in shaping the tumor-stroma crosstalk. J Mol Cell Biol 11(7):600–604 31318969 10.1093/jmcb/mjz071 PMC6736352

[36] Addadi Y, Moskovits N, Granot D et al (2010) p53 status in stromal fibroblasts modulates tumor growth in an SDF1-dependent manner. Cancer Res 70(23):9650–9658 20952507 10.1158/0008-5472.CAN-10-1146 PMC2999653

[37] Cordani M, Pacchiana R, Butera G et al (2016) Mutant p53 proteins alter cancer cell secretome and tumour microenvironment: involvement in cancer invasion and metastasis. Cancer Lett 376(2):303–309 27045472 10.1016/j.canlet.2016.03.046

[38] McCubrey JA, Yang LV, Abrams SL et al (2022) Effects of TP53 mutations and miRs on immune responses in the tumor microenvironment important in Pancreatic cancer progression. Cells 11(14):2155 35883598 10.3390/cells11142155 PMC9318640

[39] Farhood B, Najafi M, Mortezaee K (2019) CD8(+) cytotoxic T lymphocytes in cancer immunotherapy: a review. J Cell Physiol 234(6):8509–8521 30520029 10.1002/jcp.27782

[40] Wang W, Green M, Choi JE et al (2019) CD8(+) T cells regulate tumour ferroptosis during cancer immunotherapy. Nature 569(7755):270–274 31043744 10.1038/s41586-019-1170-y PMC6533917

[41] Golstein P, Griffiths GM (2018) An early history of T cell-mediated cytotoxicity. Nat Rev Immunol 18(8):527–535 29662120 10.1038/s41577-018-0009-3

[42] Ben Safta T, Ziani L, Favre L et al (2015) Granzyme B-activated p53 interacts with Bcl-2 to promote cytotoxic lymphocyte-mediated apoptosis. J Immunol 194(1):418–428 25404359 10.4049/jimmunol.1401978

[43] Thiery J, Safta TB, Ziani L et al (2015) Mechanisms of cytotoxic lymphocyte-mediated apoptosis and relationship with the tumor suppressor p53. Crit Rev Immunol 35(6):433–449 27279042 10.1615/CritRevImmunol.2016015691

[44] Di Minin G, Bellazzo A, Dal Ferro M et al (2014) Mutant p53 reprograms TNF signaling in cancer cells through interaction with the tumor suppressor DAB2IP. Mol Cell 56(5):617–629 25454946 10.1016/j.molcel.2014.10.013

[45] Yue X, Wu F, Li Y et al (2020) Gain of function mutant p53 protein activates AKT through the Rac1 signaling to promote tumorigenesis. Cell Cycle 19(11):1338–1351 32275841 10.1080/15384101.2020.1749790 PMC7469461

[46] Sallman DA, McLemore AF, Aldrich AL et al (2020) TP53 mutations in myelodysplastic syndromes and secondary AML confer an immunosuppressive phenotype. Blood 136(24):2812–2823 32730593 10.1182/blood.2020006158 PMC7731792

[47] Buchan SL, Rogel A, Al-Shamkhani A (2018) The immunobiology of CD27 and OX40 and their potential as targets for cancer immunotherapy. Blood 131(1):39–48 29118006 10.1182/blood-2017-07-741025

[48] Amatore F, Gorvel L, Olive D (2018) Inducible co-stimulator (ICOS) as a potential therapeutic target for anti-cancer therapy. Expert Opin Ther Targets 22(4):343–351 29468927 10.1080/14728222.2018.1444753

[49] Melaiu O, Lucarini V, Cifaldi L et al (2019) Influence of the tumor microenvironment on NK cell function in solid tumors. Front Immunol 10:3038 32038612 10.3389/fimmu.2019.03038 PMC6985149

[50] Sivori S, Pende D, Quatrini L et al (2021) NK cells and ILCs in tumor immunotherapy. Mol Aspects Med 80:100870 32800530 10.1016/j.mam.2020.100870

[51] Duan S, Guo W, Xu Z et al (2019) Natural killer group 2D receptor and its ligands in cancer immune escape. Mol Cancer 18(1):29 30813924 10.1186/s12943-019-0956-8 PMC6391774

[52] Veneziani I, Fruci D, Compagnone M et al (2019) The BET-bromodomain inhibitor JQ1 renders neuroblastoma cells more resistant to NK cell-mediated recognition and killing by downregulating ligands for NKG2D and DNAM-1 receptors. Oncotarget 10(22):2151–2160 31040907 10.18632/oncotarget.26736 PMC6481332

[53] Uddin MB, Roy KR, Hill RA et al (2022) p53 missense mutant G242A subverts natural killer cells in sheltering mouse breast cancer cells against immune rejection. Exp Cell Res 417(1):113210 35597298 10.1016/j.yexcr.2022.113210

[54] Pan Y, Yu Y, Wang X et al (2020) Tumor-associated macrophages in tumor immunity. Front Immunol 11:583084 33365025 10.3389/fimmu.2020.583084 PMC7751482

[55] Boutilier AJ, Elsawa SF (2021) Macrophage polarization states in the tumor microenvironment. Int J Mol Sci 22(13):6995 34209703 10.3390/ijms22136995 PMC8268869

[56] Wu K, Lin K, Li X et al (2020) Redefining tumor-associated macrophage subpopulations and functions in the tumor microenvironment. Front Immunol 11:1731 32849616 10.3389/fimmu.2020.01731 PMC7417513

[57] Shi X, Kaller M, Rokavec M et al (2020) Characterization of a p53/miR-34a/CSF1R/STAT3 feedback loop in colorectal cancer. Cell Mol Gastroenterol Hepatol 10(2):391–418 32304779 10.1016/j.jcmgh.2020.04.002 PMC7423584

[58] Fang H, Huang Y, Luo Y et al (2022) SIRT1 induces the accumulation of TAMs at colorectal cancer tumor sites via the CXCR4/CXCL12 axis. Cell Immunol 371:104458 34847407 10.1016/j.cellimm.2021.104458

[59] Cooks T, Pateras IS, Jenkins LM et al (2018) Mutant p53 cancers reprogram macrophages to tumor supporting macrophages via exosomal miR-1246. Nat Commun 9(1):771 29472616 10.1038/s41467-018-03224-w PMC5823939

[60] Wellenstein MD, Coffelt SB, Duits DEM et al (2019) Loss of p53 triggers WNT-dependent systemic inflammation to drive breast cancer metastasis. Nature 572(7770):538–542 31367040 10.1038/s41586-019-1450-6 PMC6707815

[61] Tian X, Shen H, Li Z et al (2019) Tumor-derived exosomes, myeloid-derived suppressor cells, and tumor microenvironment. J Hematol Oncol 12(1):84 31438991 10.1186/s13045-019-0772-z PMC6704713

[62] Yang Y, Zhang M, Zhang Y et al (2023) 5-Fluorouracil suppresses colon tumor through activating the p53-Fas pathway to sensitize myeloid-derived suppressor cells to FasL(+) cytotoxic T lymphocyte cytotoxicity. Cancers 15(5):1563 36900354 10.3390/cancers15051563 PMC10001142

[63] Li L, Xu J, Qiu G et al (2018) Epigenomic characterization of a p53-regulated 3p22.2 tumor suppressor that inhibits STAT3 phosphorylation via protein docking and is frequently methylated in esophageal and other carcinomas. Theranostics 8(1):61–77 29290793 10.7150/thno.20893 PMC5743460

[64] Guha P, Gardell J, Darpolor J et al (2019) STAT3 inhibition induces Bax-dependent apoptosis in liver tumor myeloid-derived suppressor cells. Oncogene 38(4):533–548 30158673 10.1038/s41388-018-0449-z

[65] Chang LC, Chiang SK, Chen SE et al (2018) Heme oxygenase-1 mediates BAY 11–7085 induced ferroptosis. Cancer Lett 416:124–137 29274359 10.1016/j.canlet.2017.12.025

[66] Zhu H, Klement JD, Lu C et al (2021) Asah2 represses the p53-Hmox1 axis to protect myeloid-derived suppressor cells from ferroptosis. J Immunol 206(6):1395–1404 33547170 10.4049/jimmunol.2000500 PMC7946776

[67] Xu QR, Tang J, Liao HY et al (2021) Long non-coding RNA MEG3 mediates the miR-149-3p/FOXP3 axis by reducing p53 ubiquitination to exert a suppressive effect on regulatory T cell differentiation and immune escape in esophageal cancer. J Transl Med 19(1):264 34140005 10.1186/s12967-021-02907-1 PMC8212454

[68] Yang P, Li QJ, Feng Y et al (2012) TGF-β-miR-34a-CCL22 signaling-induced Treg cell recruitment promotes venous metastases of HBV-positive hepatocellular carcinoma. Cancer Cell 22(3):291–303 22975373 10.1016/j.ccr.2012.07.023 PMC3443566

[69] Biffi G, Tuveson DA (2021) Diversity and biology of cancer-associated fibroblasts. Physiol Rev 101(1):147–176 32466724 10.1152/physrev.00048.2019 PMC7864232

[70] Liu Q, Yu B, Tian Y et al (2020) P53 mutant p53(N236S) regulates cancer-associated fibroblasts properties through Stat3 pathway. Onco Targets Ther 13:1355–1363 32104002 10.2147/OTT.S229065 PMC7027832

[71] Cui Y, Guo G (2016) Immunomodulatory function of the tumor suppressor p53 in host immune response and the tumor microenvironment. Int J Mol Sci 17(11):1942 27869779 10.3390/ijms17111942 PMC5133937

[72] Fang Z, Meng Q, Xu J et al (2023) Signaling pathways in cancer-associated fibroblasts: recent advances and future perspectives. Cancer Commun 43(1):3–41 10.1002/cac2.12392 PMC9859735 36424360

[73] Oh IR, Raymundo B, Kim M et al (2020) Mesenchymal stem cells co-cultured with colorectal cancer cells showed increased invasive and proliferative abilities due to its altered p53/TGF-β1 levels. Biosci Biotechnol Biochem 84(2):256–267 31601153 10.1080/09168451.2019.1676692

[74] Lin SY, Dolfi SC, Amiri S et al (2013) P53 regulates the migration of mesenchymal stromal cells in response to the tumor microenvironment through both CXCL12-dependent and -independent mechanisms. Int J Oncol 43(6):1817–1823 24064862 10.3892/ijo.2013.2109 PMC3834256

[75] Velletri T, Xie N, Wang Y et al (2016) P53 functional abnormality in mesenchymal stem cells promotes osteosarcoma development. Cell Death Dis 7(1):e2015 26775693 10.1038/cddis.2015.367 PMC4816167

[76] Hardwick NR, Frankel P, Ruel C et al (2018) p53-Reactive T cells are associated with clinical benefit in patients with platinum-resistant epithelial ovarian cancer after treatment with a p53 vaccine and gemcitabine chemotherapy. Clin Cancer Res 24(6):1315–1325 29301826 10.1158/1078-0432.CCR-17-2709 PMC5856606

[77] van de Donk N, Zweegman S (2023) T-cell-engaging bispecific antibodies in cancer. Lancet 402(10396):142–158 37271153 10.1016/S0140-6736(23)00521-4

[78] Hsiue EH, Wright KM, Douglass J et al (2021) Targeting a neoantigen derived from a common TP53 mutation. Science 371(6533):10 33649166 10.1126/science.abc8697 PMC8208645

[79] Shi D, Jiang P (2021) A different facet of p53 function: regulation of immunity and inflammation during tumor development. Front Cell Dev Biol 9:762651 34733856 10.3389/fcell.2021.762651 PMC8558413

[80] Chasov V, Zaripov M, Mirgayazova R et al (2021) Promising new tools for targeting p53 mutant cancers: humoral and cell-based immunotherapies. Front Immunol 12:707734 34484205 10.3389/fimmu.2021.707734 PMC8411701

[81] García-Garrido E, Cordani M, Somoza Á (2021) Modified gold nanoparticles to overcome the chemoresistance to gemcitabine in mutant p53 cancer cells. Pharmaceutics 13(12):2067 34959348 10.3390/pharmaceutics13122067 PMC8703659

[82] Ma J, Murphy M, O’Dwyer PJ et al (2002) Biochemical changes associated with a multidrug-resistant phenotype of a human glioma cell line with temozolomide-acquired resistance. Biochem Pharmacol 63(7):1219–1228 11960598 10.1016/s0006-2952(02)00876-6

[83] Fruehauf JP, Brem H, Brem S et al (2006) In vitro drug response and molecular markers associated with drug resistance in malignant gliomas. Clin Cancer Res 12(15):4523–4532 16899598 10.1158/1078-0432.CCR-05-1830

[84] Srivenugopal KS, Shou J, Mullapudi SR et al (2001) Enforced expression of wild-type p53 curtails the transcription of the O(6)-methylguanine-DNA methyltransferase gene in human tumor cells and enhances their sensitivity to alkylating agents. Clin Cancer Res 7(5):1398–1409 11350911

[85] Kim SS, Rait A, Kim E et al (2014) A nanoparticle carrying the p53 gene targets tumors including cancer stem cells, sensitizes glioblastoma to chemotherapy and improves survival. ACS Nano 8(6):5494–5514 24811110 10.1021/nn5014484 PMC4076028

[86] Kong N, Tao W, Ling X et al (2019) Synthetic mRNA nanoparticle-mediated restoration of p53 tumor suppressor sensitizes p53-deficient cancers to mTOR inhibition. Sci Transl Med. 10.1126/scitranslmed.aaw1565 31852795 10.1126/scitranslmed.aaw1565 PMC7024563

[87] Prabha S, Sharma B, Labhasetwar V (2012) Inhibition of tumor angiogenesis and growth by nanoparticle-mediated p53 gene therapy in mice. Cancer Gene Ther 19(8):530–537 22595792 10.1038/cgt.2012.26 PMC3400709

[88] Mei Y, Qin X, Yang Z et al (2024) Engineered a dual-targeting HA-TPP/A nanoparticle for combination therapy against KRAS-TP53 co-mutation in gastrointestinal cancers. Bioact Mater 32:277–291 37876556 10.1016/j.bioactmat.2023.10.003 PMC10590736

[89] Koo N, Sharma AK, Narayan S (2022) Therapeutics targeting p53-MDM2 interaction to induce cancer cell death. Int J Mol Sci 23(9):5005 35563397 10.3390/ijms23095005 PMC9103871

[90] Zhou X, Singh M, Sanz Santos G et al (2021) Pharmacologic activation of p53 triggers viral mimicry response thereby abolishing tumor immune evasion and promoting antitumor immunity. Cancer Discov 11(12):3090–3105 34230007 10.1158/2159-8290.CD-20-1741 PMC9414294

[91] Han Y, Liu D, Li L (2020) PD-1/PD-L1 pathway: current researches in cancer. Am J Cancer Res 10(3):727–742 32266087 PMC7136921

[92] Wang HQ, Mulford IJ, Sharp F et al (2021) Inhibition of MDM2 promotes antitumor responses in p53 wild-type cancer cells through their interaction with the immune and stromal microenvironment. Cancer Res 81(11):3079–3091 33504557 10.1158/0008-5472.CAN-20-0189

[93] Xiao Y, Chen J, Zhou H et al (2022) Combining p53 mRNA nanotherapy with immune checkpoint blockade reprograms the immune microenvironment for effective cancer therapy. Nat Commun 13(1):758 35140208 10.1038/s41467-022-28279-8 PMC8828745

[94] Shouval R, Alarcon Tomas A, Fein JA et al (2022) Impact of TP53 genomic alterations in large B-cell lymphoma treated with CD19-chimeric antigen receptor T-cell therapy. J Clin Oncol 40(4):369–381 34860572 10.1200/JCO.21.02143 PMC8797602

[95] Zhu G, Pan C, Bei JX et al (2020) Mutant p53 in cancer progression and targeted therapies. Front Oncol 10:595187 33240819 10.3389/fonc.2020.595187 PMC7677253

[96] Fiorini C, Cordani M, Padroni C et al (2015) Mutant p53 stimulates chemoresistance of pancreatic adenocarcinoma cells to gemcitabine. Biochim Biophys Acta 1853(1):89–100 25311384 10.1016/j.bbamcr.2014.10.003

[97] Dong ZY, Zhong WZ, Zhang XC et al (2017) Potential predictive value of TP53 and KRAS mutation status for response to PD-1 blockade immunotherapy in lung adenocarcinoma. Clin Cancer Res 23(12):3012–3024 28039262 10.1158/1078-0432.CCR-16-2554

[98] Li Y, Zhang MC, Xu XK et al (2019) Functional diversity of p53 in human and wild animals. Front Endocrinol 10:152 10.3389/fendo.2019.00152 PMC6422910 30915036

[99] Dong P, Xiong Y, Yu J et al (2018) Control of PD-L1 expression by miR-140/142/340/383 and oncogenic activation of the OCT4-miR-18a pathway in cervical cancer. Oncogene 37(39):5257–5268 29855617 10.1038/s41388-018-0347-4 PMC6160397

[100] D’Orazi G, Cordani M, Cirone M (2021) Oncogenic pathways activated by pro-inflammatory cytokines promote mutant p53 stability: clue for novel anticancer therapies. Cell Mol Life Sci 78(5):1853–1860 33070220 10.1007/s00018-020-03677-7 PMC11072129

[101] Xiu W, Ma J, Lei T et al (2018) AG490 reverses phenotypic alteration of dendritic cells by bladder cancer cells. Oncol Lett 16(3):2851–2856 30127871 10.3892/ol.2018.9028 PMC6096164

[102] Groot J, Ott M, Wei J et al (2022) A first-in-human phase I trial of the oral p-STAT3 inhibitor WP1066 in patients with recurrent malignant glioma. CNS Oncol 11(2):Cns87 35575067 10.2217/cns-2022-0005 PMC9134932

[103] Cai Z, Wang J, Li Y et al (2023) Overexpressed Cyclin D1 and CDK4 proteins are responsible for the resistance to CDK4/6 inhibitor in breast cancer that can be reversed by PI3K/mTOR inhibitors. Sci China Life Sci 66(1):94–109 35982377 10.1007/s11427-021-2140-8

[104] Goldman JW, Rosen LS, Tolcher AW et al (2018) Phase 1 and pharmacokinetic study of LY3007113, a p38 MAPK inhibitor, in patients with advanced cancer. Invest New Drugs 36(4):629–637 29196957 10.1007/s10637-017-0532-2 PMC6061137

[105] Talaei S, Mellatyar H, Asadi A et al (2019) Spotlight on 17-AAG as an Hsp90 inhibitor for molecular targeted cancer treatment. Chem Biol Drug Des 93(5):760–786 30697932 10.1111/cbdd.13486

[106] Carlsen L, Zhang S, Tian X et al (2023) The role of p53 in anti-tumor immunity and response to immunotherapy. Front Mol Biosci 10:1148389 37602328 10.3389/fmolb.2023.1148389 PMC10434531

[107] Thind AS, Monga I, Thakur PK et al (2021) Demystifying emerging bulk RNA-Seq applications: the application and utility of bioinformatic methodology. Brief Bioinform. 10.1093/bib/bbab259 34329375 10.1093/bib/bbab259

[108] Lu J, Sheng Y, Qian W et al (2023) scRNA-seq data analysis method to improve analysis performance. IET Nanobiotechnol 17(3):246–256 36727937 10.1049/nbt2.12115 PMC10190501

[109] Asp M, Bergenstråhle J, Lundeberg J (2020) Spatially resolved transcriptomes-next generation tools for tissue exploration. BioEssays 42(10):e1900221 32363691 10.1002/bies.201900221

[110] Liu Z, Sun D, Wang C (2022) Evaluation of cell-cell interaction methods by integrating single-cell RNA sequencing data with spatial information. Genome Biol 23(1):218 36253792 10.1186/s13059-022-02783-y PMC9575221

[111] Wei R, He S, Bai S et al (2022) Spatial charting of single-cell transcriptomes in tissues. Nat Biotechnol 40(8):1190–1199 35314812 10.1038/s41587-022-01233-1 PMC9673606

[112] Sorrentino A, Menevse AN, Michels T, Volpin V, Durst FC, Sax J, Xydia M, Hussein A, Stamova S, Spoerl S, Heuschneider N, Muehlbauer J, Jeltsch KM, Rathinasamy A, Werner-Klein M, Breinig M, Mikietyn D, Kohler C, Poschke I, Purr S, Reidell O, Martins Freire C, Offringa R, Gebhard C, Spang R, Rehli M, Boutros M, Schmidl C, Khandelwal N, Beckhove P (2022) Salt-inducible kinase 3 protects tumor cells from cytotoxic T-cell attack by promoting TNF-induced NF-κB activation. J Immunother Cancer 10(5):e004258 35606086 10.1136/jitc-2021-004258 PMC9174898

